# Ganoderiol A-Enriched Extract Suppresses Migration and Adhesion of MDA-MB-231 Cells by Inhibiting FAK-SRC-Paxillin Cascade Pathway

**DOI:** 10.1371/journal.pone.0076620

**Published:** 2013-10-29

**Authors:** Guo-Sheng Wu, Yue-Lin Song, Zhi-Qi Yin, Jia-Jie Guo, Sheng-Peng Wang, Wen-Wen Zhao, Xiu-Ping Chen, Qing-Wen Zhang, Jin-Jian Lu, Yi-Tao Wang

**Affiliations:** 1 State Key Laboratory of Quality Research in Chinese Medicine, Institute of Chinese Medical Sciences, University of Macau, Macao, China; 2 Department of Natural Medicinal Chemistry, China Pharmaceutical University, Nanjing, China; Sun Yat-sen University Medical School, China

## Abstract

Cell adhesion, migration and invasion are critical steps for carcinogenesis and cancer metastasis. *Ganoderma lucidum*, also called Lingzhi in China, is a traditional Chinese medicine, which exhibits anti-proliferation, anti-inflammation and anti-metastasis properties. Herein, GAEE, *G. lucidum* extract mainly contains ganoderiol A (GA), dihydrogenated GA and GA isomer, was shown to inhibit the abilities of adhesion and migration, while have a slight influence on that of invasion in highly metastatic breast cancer MDA-MB-231 cells at non-toxic doses. Further investigation revealed that GAEE decreased the active forms of focal adhesion kinase (FAK) and disrupted the interaction between FAK and SRC, which lead to deactivating of paxillin. Moreover, GAEE treatment downregulated the expressions of RhoA, Rac1, and Cdc42, and decreased the interaction between neural Wiskott-Aldrich Syndrome protein (N-WASP) and Cdc42, which impair cell migration and actin assembly. To our knowledge, this is the first report to show that *G.lucidum* triterpenoids could suppress cell migration and adhesion through FAK-SRC-paxillin signaling pathway. Our study also suggests that GAEE may be a potential agent for treatment of breast cancer.

## Introduction

Breast cancer is the second most common form of cancer worldwide and the leading cause of cancer death among women [1]. Although the significant improvements in preventing and diagnosis of this diseases by increasing public health education, pre-clinical screening, and targeted therapies [2], [3], the mortality remains relatively high because of the recurrence and distant metastases [4], [5]. Thus, screening novel compounds targeting tumor metastasis is an effective way to eradicate this high-invasive cancer.

The metastatic process includes the loss of cell-to-cell and cell-to-basement-membrane adhesion, epithelial-mesenchymal transition, increased cell motility and invading extracellular matrix (ECM) [6]. Studies of cancer metastasis have traditionally focused on the migration and adhesion of individual cancer cells and the regulatory molecules involved.

FAK, a non-receptor protein tyrosine kinase, has been implicated in controlling integrin- and growth factor receptor-mediated biological processes including cell spreading, motility, migration, differentiation, angiogenesis and survival [7]. FAK regulates cell adhesion and migration mainly by its auto-phosphorylation at Tyr 397 and creating binding site for SRC. The FAK/SRC complex subsequently phosphorylates other sites of FAK thereby triggering activation of cell migration signal pathways. Furthermore, FAK functions as a scaffolding protein associating with P130Cas, paxillin and other adaptors to promote cell migration and adhesion [8]. Increased FAK expression and activity are correlated with malignant or metastatic disease and poor prognosis in patients [9].

Paxillin is an intracellular scaffold protein, which recruits structural or regulatory proteins to modulate the dynamic changes and cytoskeletal reorganization during cell adhesion and migration [10]. Paxillin associates with many proteins to regulate cell cytoskeleton formation and tumor metastasis. Among these associations, interaction between FAK and paxillin is critical for the activation of signaling implicated in the control of cell motility [11], [12]. FAK/SRC complex activates paxillin via phosphorylation at tyrosine residues 118. Upon activation, multiple signaling pathways can coordinate to modulate cytoskeleton and promote the activities of Rho GTPases that ultimately facilitate cell migration [13].

Rho GTPases represent a widely expressed and evolutionarily conserved family of small GTP-binding proteins involved in actin dynamics, migration, and proliferation. Among the Rho GTPases, RhoA, Rac1 and Cdc42 have been well studied and characterized. They play critical roles in cancer progression and tumor metastasis, including cell cycle control, cell polarity formation, cell migration, cell invasion and angiogenesis. Over expression of Rho GTPase genes and enhanced Rho GTPase activities are often found in tumor cells and associated with increased cancer metastasis [14].

N-WASP is a key regulator of actin cytoskeleton and a component of invadopodia in metastatic cancer cells. Binding of Cdc42 to N-WASP leads to a conformational alternation of N-WASP, which subsequently promotes the interaction between actin-related protein 2 and 3 (Arp2/3) and N-WASP, and finally initiates actin assembly [15]. N-WASP-mediated actin nucleation contributes to plasma membrane protrusions and is linked with cell invasion and migration [16].

*Ganoderma lucidum*, a Chinese medicinal herb, is considered to possess the ability of promoting health and increasing life expectancy in East Asia countries. Our previous studies have demonstrated that the triterpenoids isolated from *G. lucidum* inhibit breast cancer cell proliferation by retarding cell cycle in G1 phase and inducing apoptosis [17], [18]. *G. lucidum* triterpenoids (mainly refer to ganoderic acids or lucidenic acids) also suppress breast cancer invasion by decreasing expression and enzyme activities of matrix metalloproteinases (MMPs) and urokinase plaminogen activator (uPA) [19]. In addition, repression of NF-κB transcriptional activity is parallel to *G. lucidum* water extract-induced cancer cell migration inhibition [20]. In the present study, GAEE, a *G. lucidum* triterpenoids extract mainly contains GA, dihydrogenated GA and GA isomer (>95%), was used to examine the anti-metastatic effects in human highly invasive breast cancer MDA-MB-231 cells. GAEE suppressed cell migration and adhesion in a concentration-dependent manner mainly due to blocking SRC/FAK-associated signaling pathway and downregulation of p-paxillin (Y118). Furthermore, inhibiting the expression of Rho GTPases and disruption the interaction between N-WASP and Cdc42 by GAEE also contributed to its anti-migration and anti-adhesion effects.

## Materials and Methods

### Preparation and characterization of GAEE

**Figure 1:**
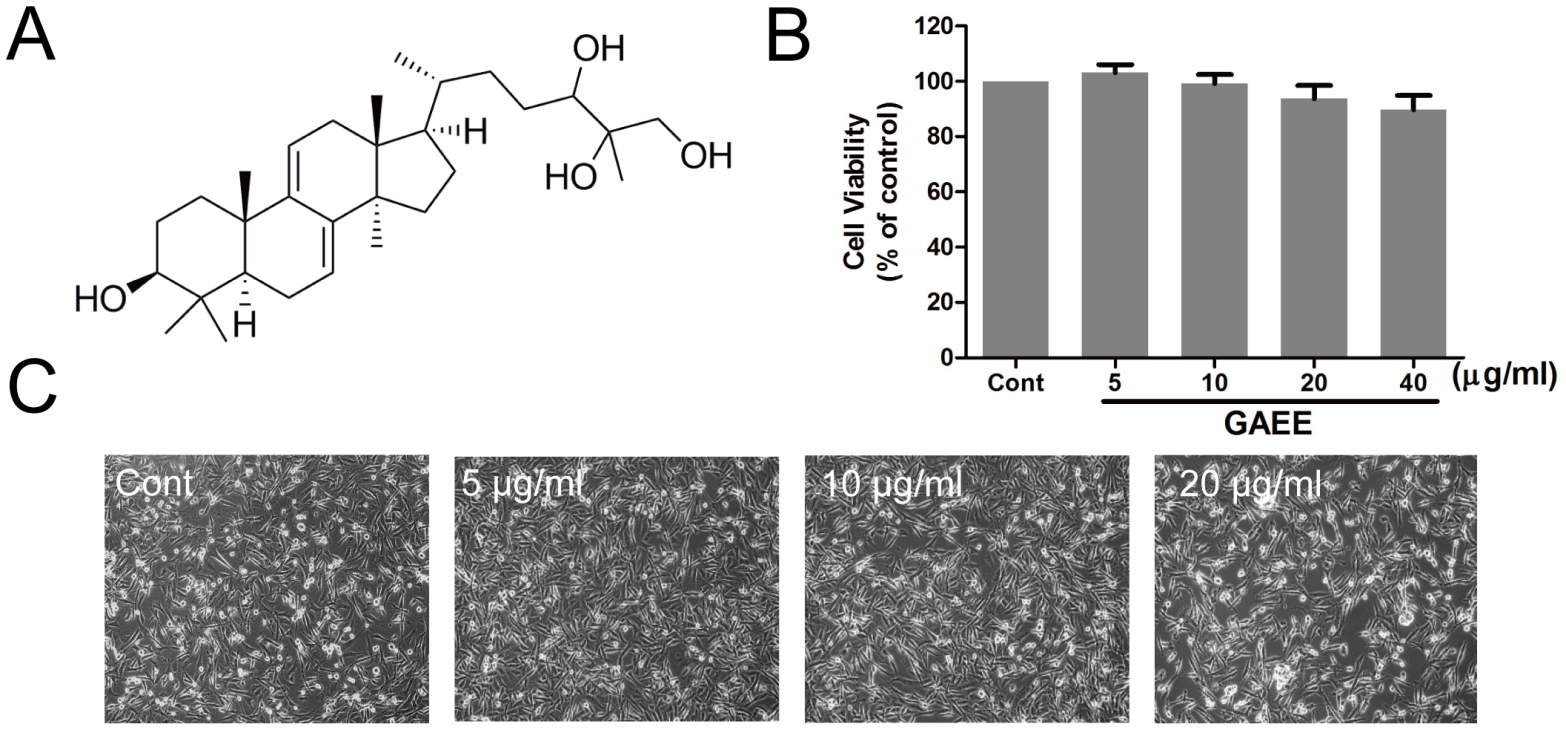
Effects of GAEE on cell viability in MDA-MB-231 cells. (A) The chemical structure of ganoderiol A; (B) MDA-MB-231 cells were treated with GAEE for 24 h and the cell viability was tested using MTT assay. Values are expressed as the mean ± SEM of seven independent experiments. (C) The morphological observations of MDA-MB-231 cells after GAEE treatment. Cells were treated with various concentrations of GAEE for 24 h and the photos were taken with an AxioCam HRC CCD phase contrast microscope.

The air-dried fruiting bodies of *G. lucidum* (100 kg) were smashed and extracted with 95% (v/v) aqueous ethanol under heating reflux (1000 L×3, 2 h each). The extract was concentrated under vacuum to remove solvent, and then the residues were suspended with H_2_O and partitioned successively by petroleum ether, ethyl acetate, and *n*-butanol. The ethyl acetate extract (370 g) was subjected onto column chromatography (CC) over silica gel using gradient mixtures of CH_2_Cl_2_-MeOH (100∶0-50∶50) as eluent to afford seven fractions. GAEE (40 mg) was yielded by silica gel CC eluting with gradient mixtures of CH_2_Cl_2_-MeOH (100∶2-100∶60) repeatedly followed by Sephadex LH-20 column chromatography (CHCl_3_-MeOH, 1∶1). Then GAEE was qualitatively analyzed with a high performance liquid chromatography coupled with tandem mass spectrometry (LC-MS/MS) system which consisted of a Agilent 1200SL HPLC system (Agilent Technologies, USA) equipped with an Agilent Zorbax SB-C18 column (100 mm×2.1 mm i.d., particle size 3.5 µm) and an ESI mass spectrometer (ABSciex API 4000Q-trap Mass spectrometer, Forster City, CA, USA), and GAEE was identified to be a mixture mainly containing GA (approximate 70% of the whole fraction, **Fig. 1A**), dihydrogenated GA and GA isomer. GAEE was dissolved in dimethyl sulfoxide, and stored at −20°C until use.

### Cell culture

The human breast cancer MDA-MB-231 cells (ATCC, Rockville, MD) were cultured in DMEM medium supplemented with 10% fetal bovine serum (GIBCO BRL, Carlsbad, CA), 100 U/ml penicillin and 100 µg/ml streptomycin, and grown in a 37°C incubator.

### Observation of morphologic changes

MDA-MB-231 cells were seeded into 6-well plates and treated with indicated concentration of GAEE for 24 h. The cellular morphology was observed and photographed with AxioCam HRC CCD camera (Carl Zeiss).

### Hoechst-33342 staining

After treatment with GAEE for 24 h, cells were washed with PBS and fixed with 4% formaldehyde in PBS. Cells were then stained for 15–30 min using 10 µg/ml Hoechst-33342. After washing for three times, nuclear staining was examined by Axiovert 200 fluorescent inverted microscope (Carl Zeiss) and typical images were captured using AxioCam HRC CCD camera (Carl Zeiss).

### MTT assay

Exponentially growing MDA-MB-231 cells were planted into 96-well plates and after adhesion, the cells were treated with various concentration of GAEE. The cell viability was determined after 24 h-incubation by adding 20 µl MTT (5 mg/ml) (Molecular Probes, Eugene, OR). Then the MTT-containing medium was aspirated slightly after 4 h, and 100 µl dimethyl sulfoxide was added to solubilize the formazan followed by shaking 10 min under the dark. The absorbance at 570 nm was recorded using a Multilabel counter (Perkin Elmer, Singapore).

### Cell cycle analysis

MDA-MB-231 cells seeded into 6-well plates were treated with series concentrations of GAEE for indicated time. Cells were harvested and fixed in 70% ethanol and then stored at 4°C overnight. Cells were stained in PBS containing 5 µg/ml RNase and 20 µg/ml propidium iodide in the dark at room temperature for 30 min and analyzed using a flow cytometry (Becton Dickinson FACS Canto, Franklin Lakes, NJ). At least 10,000 events were counted for each sample. The DNA content in the G1, S, and G2/M phases was analyzed using ModFit 161 LT version 3.0 software (Verity Software House, Topsham, USA).

### Transwell assay

The invasion and migration of MDA-MB-231 were assayed in Transwell chambers according to the methods previously reported with slight modifications [21]. Briefly, The upper chamber of a Transwell insert (8 µm pore size) was coated with 100 µl 1∶6 mixture of Matrigel (BD Biosciences, Bedford, MA) : PBS (invasion assay) or 100 µl PBS (migration assay) and dried for 30 min at 37°C. The lower chamber was filled 500 µl DMEM with 10% FBS. MDA-MB-231cells were treated with series concentration of GAEE for 24 h. After treatment, the cells were suspended and reseeded into upper chamber in serum-free media at a density of 1×10^5^ cells per well. After 24 h incubation, cells on the top aspect of the membrane were removed by cotton swabs, and cells that invaded on the lower surface of the membrane were fixed using 4% paraformaldehyde (PFA) and stained with crystal violet (Beyotime Institute of Biotechnology, China). Random fields were counted and the typical images were photographed by AxioCam HRC CCD camera (Carl Zeiss).

### Wound-healing assay

MDA-MB-231 cells were seeded into 12-well plates with complete medium and grown to confluence. Then, the monolayer was scraped by 200 µl micropipette tip and washed with PBS to remove the floating cells. Medium with GAEE at indicated concentrations was added to cultures. Photomicrographs were taken at 0 h and 24 h with Axiovert 200 inverted microscope (Carl Zeiss) and AxioCam HRC CCD camera (Carl Zeiss). Some representative fields were photographed.

### Cell adhesion assay

Cell adhesion assay was performed as reported previously with modifications [22]. MDA-MB-231 cells were incubated in the presence or absence of GAEE for 24 h. Cells were harvested, counted and resuspended in culture medium. The cells were then transferred to a 96-well plate precoated with Matrigel (25 µg/ml). After incubation for 2 h at 37°C, the medium was discarded and washed with PBS to remove the non-adherent cells. Then some wells were fixed with 4% (w/v) formaldehyde, stained with 0.2% crystal violet and photographed. The other wells were quantified by MTT assay to determine the number of adherent cells.

### Western blot analysis

Cells were lysed in the lysis buffer. The proteins of the lysates were quantified with BCA Protein Assay Kit (Pierce, Rockford, IL). 50 µg of total proteins were subjected to 6–12% SDS-PAGE and transferred onto PVDF membranes, blocked with 5% nonfat milk in TBST (20 mM Tris, 500 mM NaCl, and 0.1% Tween-20) at room temperature for 2 h with rocking. The membranes were probed with specific primary antibodies against caspase-9, Bcl-2, Bax, PARP, FAK, p-FAK (Y395), p-FAK (Y925), SRC, p-SRC (Y527), p-SRC (Y416), integrin β1, integrin β4, paxillin, p-paxillin (Y118), Rac1, RhoA, Cdc42, β-action, and GAPDH (Cell Signaling Technology, Beverly, MA), N-WASP and SRC (Santa Cruz Biotechnology, Santa Cruz, CA) overnight at 4°C. After washing with TBST three times for 15 min each, the membranes were incubated with horseradish-peroxidase-conjugated secondary antibodies (Santa Cruz Biotechnology, Santa Cruz, CA) in TBST at room temperature for 1 h. After washing three times in TBST for 15 min, the specific protein bands were visualized using an ECL advanced Western blot detection kit (Amersham Life Sciences, GE Healthcare, NJ). Equal protein loading was verified by probing with anti-β-actin or GAPDH antibodies. Quantification was performed using ImageJ software.

### Immunofluorescence

Cells were seeded in 96-well plate and treated with GAEE for 24 h. After incubation, cells were fixed with 4% formaldehyde (Sigma, Germany) in PBS at 37°C for 30 min, washed with PBS, and then permeabilized with 0.5% Triton X-100 in PBS for 20 min at room temperature. Cells were washed in a blocking solution consisting of 5% BSA and 0.2% Triton X-100 and stored in the blocking solution at 4°C till labeling. For labeling, fixed cells were incubated for 2 h at 37°C with specific antibodies against to p-paxillin (Y118) in the blocking solution, followed by three washes in blocking solution. Cells were incubated with Rhodamine-conjugated goat anti-rabbit IgG in the blocking solution at 37°C for 1 h. After three washes, cells were incubated for 10 min at room temperature with Hoechst-33342 (10 µg/ml) (Molecular Probes, Eugene, OR). Cells were mounted on the stage of the In Cell Analyzer 2000 (GE Healthcare) after another three washes in PBS. The typical images were acquired.

### Immunoprecipitations

Cells were washed with cold PBS twice and lysed in cell lysis buffer for Western and IP (Beyotime Institute of Biotechnology, China) for 10 min on ice. Then the mixture was centrifuge with 13,000 g for 10 min at 4°C. The supernatant was collected, protein concentration was quantified at 1 mg/ml and 0.6 mg of protein was incubated with 4 µg of primary target antibodies against FAK, SRC (Cell Signaling Technology, Beverly, MA) and N-WASP (Santa Cruz Biotechnology, Santa Cruz, CA) or nonspecific antibody for 10 h at 4°C. Then lysates containing protein-antibody complexes were incubated with 40 µl of Protein G-agarose (Santa Cruz Biotechnology, Santa Cruz, CA) for 6 h at 4°C. Lysates were centrifuged and agarose-bound immunoprecipitated complexes were washed 6 times with cell lysis buffer. And finally beads were suspended in the loading buffer, boiled, and analyzed by SDS-PAGE.

### Statistical analysis

Data were presented as mean ± SEM. Significance was analyzed using one-way analysis of variance (ANOVA) and Tukey's multiple comparison test by GraphPad Prism (Demo, Version 5). Difference was considered significant where *P*<0.05.

## Results

### Cytotoxicity of GAEE in MDA-MB-231 cells

The effects of GAEE (0–40 µg/ml) on cell viability was detected by MTT assay and the morphological changes were observed using phase-contrast microscopy. As shown in **Fig. 1B**, cell viability was not significantly affected in GAEE-treated groups after incubation for 24 h. GAEE at a concentration between 5 µg/ml and 20 µg/ml exhibited no toxicity to MDA-MB-231 cells as obtained from the cell morphological observation (**Fig. 1C**). Thus, the concentrations used above and an incubation time of 24 h were applied in the following experiments.

### GAEE did not cause cell cycle arrest and apoptosis in MDA-MB-231 cells

**Figure 2:**
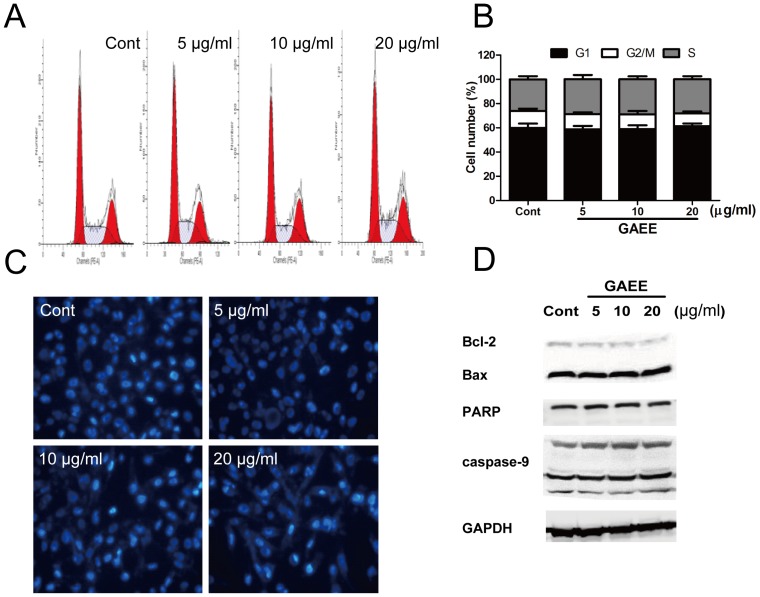
Effects of GAEE on cell cycle progression and apoptosis. (A, B) The cell cycle distribution in MDA-MB-231 cells after treatment with GAEE. Data were shown as mean ± SEM of four independent experiments. (C) Cells were exposed to various concentration of GAEE for 24 h, nuclear morphology was observed by the Hoechst-33342 staining. (D) MDA-MB-231 cells were treated with GAEE for 24 h and the protein levels of Bcl-2, Bax, PARP, and caspase-9 were examined using Western blot analysis. GAPDH was used as a loading control.

To further ascertain these doses do not affect cell growth, we performed a series of studies to measure cell cycle progression and apoptosis. As shown in **Fig. 2A** and **Fig. 2B**, GAEE (5–20 µg/ml) did not change the cell proportion in the G1, S and G2/M phases. Hoechst-33342 staining of MDA-MB-231 showed no obvious condensation of nuclear chromatin after treated with GAEE (**Fig. 2C**). Some proteins related with apoptosis were also examined by Western blot analysis. The results showed that GAEE did not affect the expression of Bcl-2, Bax, the cleavage fragment of PARP, and caspase-9 (**Fig. 2D**).

### GAEE inhibited migration, adhesion, and slightly affected invasion in MDA-MB-231 cells

**Figure 3:**
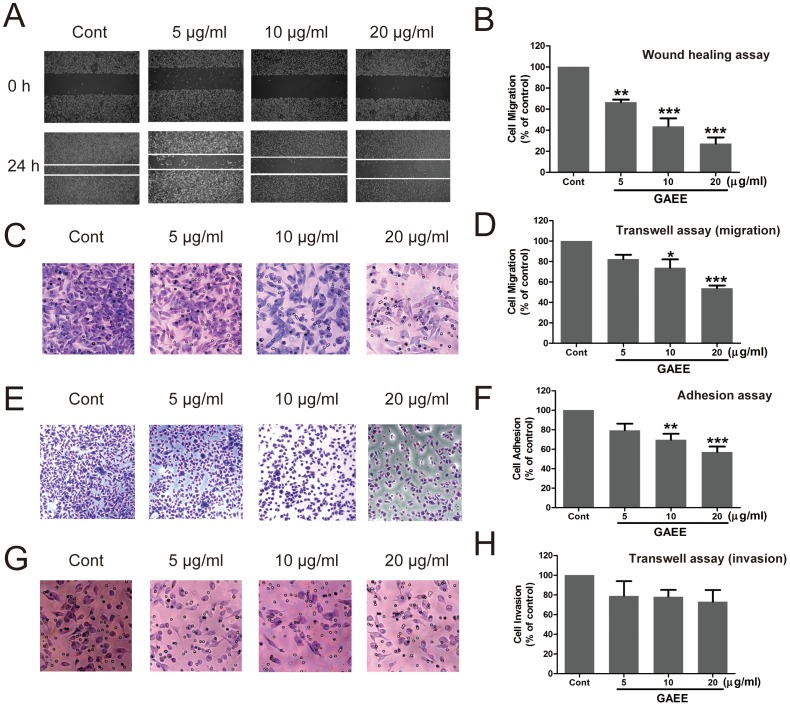
Effects of GAEE on cell migration, adhesion and invasion. (A, B) GAEE inhibited cell motility as tested by wound-healing assay in MDA-MB-231 cells. Cell monolayers were scraped using a sterile 100 microliter tip, and the cells were treated indicated concentrations of GAEE for 24 h. The migratory effects of cells were quantified by measuring the distance between the edges (White lines indicate the wound edge). (C, D) GAEE inhibited cell motility as determined by Transwell assay in MDA-MB-231 cells. The migratory cells were detected by crystal violet staining and photographed. (E, F) GAEE inhibited cell adhesion in MDA-MB-231 cells. The adhesive cells were fixed and photographed by 4% PFA and crystal violet staining. Quantitative assessment of the number of adhesive cells was performed by MTT assay. (G, H) Effect of GAEE on the invasion of MDA-MB-231 cells. In the Transwell chamber invasion assay, cells were treated with GAEE for 24 h and the invasion ability of cells was quantified by counting the number of cells that invaded the underside of the porous polycarbonate membrane using crystal violet staining. The quantification result from three independent experiments is shown on the right. All data were shown as mean ± SEM of more than triplicates compared with the untreated control. *P<0.05, **P<0.01, ***P<0.001 (one-way ANOVA with Tukey's multiple comparison test).

Migration and invasion of cancer cells are critical steps of tumor metastasis. To determine the inhibitory effect of GAEE on migration of MDA-MB-231 cells, wound-healing assay and Transwell assay (no Matrigel) were employed. As shown in **Fig. 3A** and **Fig. 3C**, GAEE concentration-dependently attenuated migration of MDA-MB-231 cells. Treatment with 5 µg/ml, 10 µg/ml and 20 µg/ml GAEE inhibited 33.4%, 56.3%, and 72.8% of cell migration, respectively, as tested by wound healing assay (**Fig. 3B**). Next, we assessed the effects of GAEE on adhesion in MDA-MB-231 cells. Compared with untreated cells, GAEE treatment decreased the number of adherent cells in a dose-dependent manner (**Fig. 3E**). In accordance with the morphological results, MTT statistical results also showed that GAEE attenuated cell adhesion after treatment with GAEE for 24 h (**Fig. 3F**). Finally, we examined the ability of cell invasion by means of Matrigel-coated Transwell assay. The results showed that treatment with GAEE slightly decreased the invasive cells but no significant decrease was observed (**Fig. 3G** and **Fig. 3H**). These data together indicate that GAEE inhibits migration and adhesion but has minimal impact on cell invasion in MDA-MB-231 cells.

### Effect of GAEE on FAK signaling

**Figure 4:**
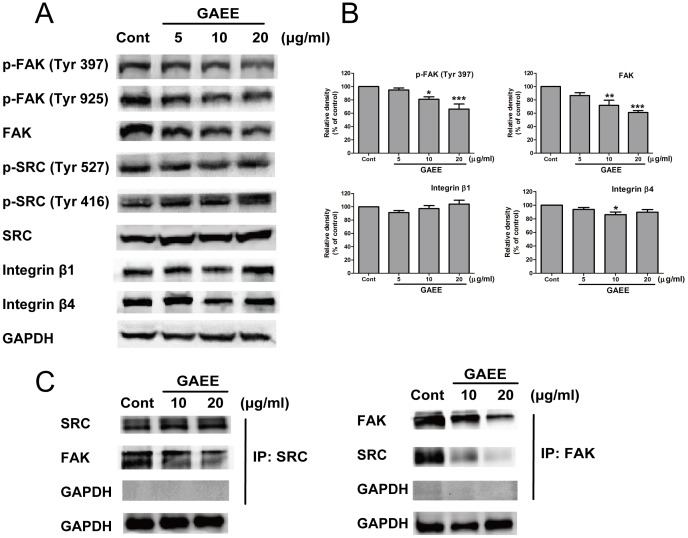
GAEE suppressed FAK signaling in MDA-MB-231 cells. (A) Immunoblots of FAK signaling proteins and other related proteins after 24 h of treatment with GAEE in MDA-MB-231 cells. Decreased expression of FAK, p-FAK (Y397), and p-FAK (Y925) were shown, while there is no alter on the expression of SRC, p-SRC, integrin β1, and integrin β4. (B) The relative densities of FAK, p-FAK (Y397), integrin β1 and integrin β4 were normalized against GAPDH by densitometric analysis. The values represented as the mean ± SEM of four independent experiments compared with control group. *P<0.05, **P<0.01, ***P<0.001 (one-way ANOVA with Tukey's multiple comparison test) (C) IP-Western confirmation for the disruption of interaction between FAK and SRC after treatment with GAEE.

Previous studies have shown that NF-κB/MMPs/uPA signaling is mainly in charge of *G. lucidum* triterpenoids-induced cell metastasis suppression. Originally, we detected this signaling after GAEE treatment but no positive trend was observed (Figure S1). Next, considering integrins and FAK play critical roles in regulation cell migration and focal adhesion formation [23], [24], we performed western blot analysis and immunoprecipitation assay to examine the activation of integrin, FAK, and SRC. Our results showed that GAEE treatment did not alter the expression levels of integrin β1 and integrin β4, but does-dependently decreased FAK and p-FAK expression (**Fig. 4A, 4B**). Integrin-ECM engagement and extracellular stimuli can activate FAK signal pathway and the full activation of FAK is benefit to its kinase activity and promoting association with other adaptors such as paxillin [25]. Herein, we found that GAEE treatment inhibited autophosporylation of FAK at Y397 and subsequent phosphorylation at Y925, but did not alter the expression of SRC and p-SRC (Y416 and Y527). Further studies revealed that GAEE decreased the formation of FAK/SRC complex, hence attenuated FAK full activation (p-FAK at Y925) and might impair downstream signaling (**Fig. 4C**).

### GAEE inhibited phosphorylation of paxillin and blocked the interaction between FAK and paxillin

**Figure 5:**
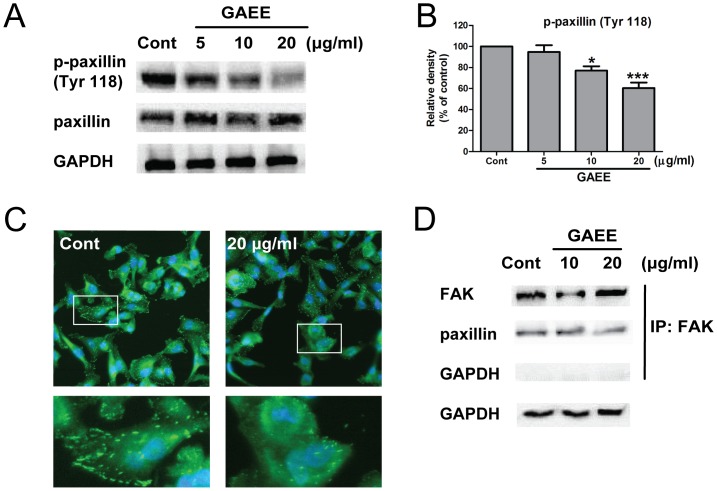
GAEE inhibited activation of paxillin in MDA-MB-231 cells. (A) Cells were treated with increasing concentrations of GAEE for 24 h, lysed, and immunoblotting with paxillin, p-paxillin (Y118). (B) The relative density of p-paxillin (Y118) was normalized against GAPDH by densitometric analysis. The values represented as the mean ±SEM of four independent experiments (*P<0.05, ***P<0.001). (C) MDA-MB-231 cells either untreated or treated with 20 µg/ml GAEE for 24 h were fixed and stained with anti-p-paxillin (Y118) antibody (green) and Hoechst 33342 (blue). Higher magnification merged image of boxed regions are shown in the lower panel. (D) The interaction between FAK and paxillin was assessed by immunoprecipitation assay after treatment with GAEE.

Paxillin is a major substrate of FAK/SRC complex to coordinate cell adhesion, cell migration and cytoskeletal reorganization [8]. Phosphorylated FAK is required for paxillin phosphorylation at Tyr 118, which is a prerequisite for cell migration [26], [27]. Recent study reported that dephosphorylation of p-paxillin (Y118) could retard cell migration even accompanied with increased expression of p-FAK (Y397), which suggests that p-paxillin (Y118) plays a pivotal role in completely activation of FAK/paxillin complex and subsequently inducing cell migration [28]. To elucidate whether FAK/SRC pathway contributes to the suppression of cell migration and adhesion via paxillin inhibition, we evaluated the activity of paxillin using western blot analysis and immunofluorescence assay. Our results showed that the expression of active form of paxillin (p-paxillin at Y118) was decreased, whereas that of total paxillin expression was not changed after GAEE treatment for 24 h (**Fig. 5A** and **Fig. 5B**). The immunofluorescence assay further confirmed the downregulation of p-paxillin after GAEE treatment (**Fig. 5C**). It has been demonstrated that paxillin phosphorylation at Y118 regulates the binding affinity between paxillin and FAK, and this association is involved in the modulation of adhesion dynamics and protrusion [29]. We further detected the association between FAK and paxillin. As shown in **Fig. 5D**, GAEE treatment attenuated the binding affinity between FAK and paxillin, which might affect cell migration and adhesion.

### Effect of GAEE on the expression of Rho GTPases and actin assembly

**Figure 6:**
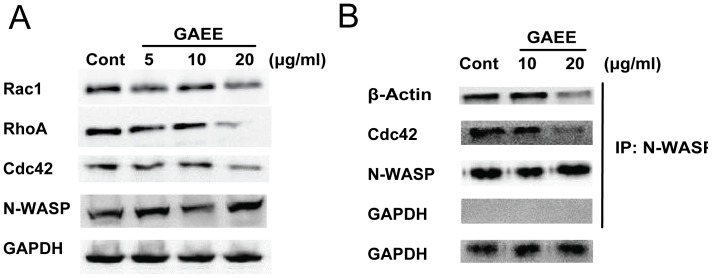
Effects of GAEE on Rho GTPases expression and actin assembly. (A) Cells were treated with indicated concentrations of GAEE for 24 h, the downregulation of RhoA, Rac1, and Cdc42 was observed, and the expression of N-WASP was not affected. (B) MDA-MB-231 cells were treated with GAEE (10 or 20 µg/ml) for 24 h, and then cell lysates were prepared and subjected to immunoprecipitation with N-WASP, and followed by immunoblotting with Cdc42 and actin. The samples were blotted with anti-N-WASP as a control.

RhoA, Rac1, and Cdc42 are critical for modulating the cell cytoskeleton reorganization and actin-associated adhesion during cell migration, and also affect cell polarity, microtubule dynamics, and membrane transport pathways [30], [31]. N-WASP is a key regulator of reorganization of actin cytoskeleton and an essential component of invadopodia [32]. During Arp2/3 complex-mediated actin ploymerization, Cdc42 binding activates N-WASP by inducing a conformational switch that initiates actin polymerization [15]. As shown in **Fig. 6A**, decrease in protein levels of Rac1, RhoA and Cdc42 was observed after GAEE treatment for 24 h, suggesting that GAEE might inhibit cell migration and adhesion through disruption cell polarity, actin assembly and focal adhesion formation. Furthermore, GAEE exposure did not alter the expression of N-WASP, but suppressed the interaction between N-WASP and Cdc42, and β-actin (**Fig. 6B**), which might cause decreased actin polymerization and disrupt the process of focal adhesion turnover during migration.

## Discussion

Numerous studies have described the importance of compounds from nature sources to treat human diseases [33]. Among these nature sources, traditional Chinese medicine is deemed as a rich reservoir of potential small chemical molecules exhibiting anticancer properties [34]. GA, a lanostane-type triterpene, was first isolated from *G.lucidum* in 1986 [35], while so far there is no systematic pharmacological research on its anticancer effect. The current study was performed to investigate the effect of GA-enriched extract on human breast cancer metastasis and elucidate the underlying mechanisms.

Tumor cell metastasis includes several steps: detachment of cancer cell from primary tumor by changing cell-cell and cell-ECM adhesion, then degrading ECM and basement membrane to migrate and invade of tumor cells into the peripheral tissue, finally intravasation into blood or lymphatic vessels and attachment at the target tissue [36]. Among these essential steps, cell adhesion, migration and invasion mainly contribute to metastatic spread. For our studies, we utilized the human breast cancer cell line MDA-MB-231 to determine the anti-metastatic effect of GAEE. This cell line is characterized as triple-negative status (estrogen receptor negative, progesterone receptor negative, and HER2 negative) and classified as the basal-like subtype of breast cancer [37]. MDA-MB-231 cell line, which originated from the most aggressive and highly metastatic breast tumors, has been generally deemed as a well-established model to screen potential anti-metastatic compounds and explore the mechanism of cancer metastasis [38]–[40]. However, only in vitro MDA-MB-231 model is not sufficient to elucidate the anti-metastatic effect of potential compounds, and in vivo assay is urgently required.

Emerging evidences have suggested that NF-κB/MMPs/uPA signaling is mainly responsible for *G. lucidum*-induced anti-invasive and anti-migratory effect [19]. Specifically, *G. lucidum* water extracts inhibit cell migration by suppressing NF-κB activity in breast and prostate cancer cells, and these inhibitory effects do not correlate with the composition and purity of herbal samples [20]. Some ganoderic acids and lucidenic acids have been reported to exert anti-invasive effect via suppression NF-κB signaling and down-regulation the expressions of MMP-9, MMP-2, uPA, and uPAR in various cancer cells [41]–[44]. Besides ganoderic acid and lucidenic acid, *G. lucidum* triterpenoids include ganoderiol, ganodsporelactone, ganoderenic acid, ganosporeric acid, ganolucidic acid, methyl ganoderate, methyl ganolucidate and so on. So far, the anti-metastatic effect of ganoderiol is rarely reported. Only a pure compound, ganodermanontriol, was found to inhibit cell adhesion, migration and invasion by decreasing the secretion of uPA and downregulation of uPAR expression in MDA-MB-231 cells [45].

Considering GA is a member of *G. lucidum* triterpenoids and probably have the similar characteristics to other *G. lucidum* triterpenoids on cancer metastasis, we performed a series of experiments to assess its effects on cell invasion and NF-κB/MMPs/uPA signaling (Figure S1). Interestingly, the cell abilities of migration and adhesion were remarkably reduced, but invasion was slightly decreased after treatment with GAEE for 24 h at non-toxic concentrations. Further studies reveled that GAEE did not affect NF-κB nuclear translocation and the expressions or activities of MMPs and uPA, which are mainly responsible for degrading ECM to facilitate cell invasion [46]. Given cell migration is a critical step of cell invasion, we suppose that the inhibitory effect of GAEE on cell migration inevitably affects invasion, hence GAEE treatment slightly suppresses cell invasion. However, in the presence of Matrigels (Transwell invasion assay), the interaction between ECM (Matrigels) and integrins might promote cell invasion signaling and counteract the GAEE induced suppression of cell migration, thus remain trigger cell invasion [47].

Generally, cell migration can be divided into five steps: lamellipodium extension at the leading edge, formation of new focal adhesions complexes, focalized proteolysis, cell body contraction and tail detachment [48]. In this study, GAEE retards the initiation and progression of cell migration by affecting at least the first two steps based on our data. Specifically, GAEE-induced downregulation of Rac1and Cdc42 might impair protrusion at the leading edge, and the inhibition of FAK activation and disruption of the interaction between FAK and SRC could impede focal adhesion turnover during migration process. In addition, considering FAK and paxillin play important roles in focal adhesion formation, which provide platforms and recruit adaptors to form focal adhesion, we conclude that GAEE decreases cell adhesion ability by FAK/SRC/paxillin pathway. Moreover, the disassociation between N-WASP and Cdc42, and downregulation of Rho GTPases impede both actin remodeling and focal adhesion, which is of benefit to the anti-migration and anti-adhesion effects of GAEE.

FAK, a non-receptor tyrosine kinase, plays a pivotal role in relaying extracellular signals from integrins to intracellular compartments by interaction with many adaptor proteins, including SRC, and paxillin among others. This scaffolding function of FAK is needed for cell motility [49], and the increased expression of FAK is correlated with carcinogenesis and tumor metastasis [30]. In addition, FAK auto-phosphorylation at Y397 facilitates the interaction between FAK and SRC, subsequently leads to full activation of FAK by phosphorylating at other tyrosine sites [25], [50]. Activated FAK/SRC complex phosphorylates paxillin at Y118 to facilitate cell adhesion and migration [8]. The idea of targeting FAK as a therapeutic strategy for cancer treatment is highly promising [51]. Herein, GAEE inhibited both FAK kinase activity and adaptor function of FAK (**Fig. 5**), which hints the potential application of GAEE as a FAK inhibitor. However, there are still questions needed to be further elucidated. First, how does GAEE downregulate the expression of FAK? NF-κB is reported to definitely participate into the regulation of FAK expression [9], while GAEE does not change the expression or translocation of NF-κB (**Fig. 4E** and **Fig. 4F**). So which transcription factors are involved in regulating the gene expression of FAK or whether GAEE enhances the degradation of FAK remains enigmatic. Second, considering FAK is an important regulator comprehensively mediated cell proliferation, cell survival and cell invasion, the key question as to how GAEE selectively inhibits FAK-induced cell migration and adhesion is unclear and needed to be further studied.

Rho GTPases, including Rac1, RhoA, and Cdc42, has been reported to cause distinct morphological changes in the actin cytoskeleton and promote cell migration [52]. Rho GTPases activities are regulated by guaninenucleotide-exchange factors (GEFs) through a cycling between an inactive (GDP-bound) and an active (GTP-bound) state. P-paxillin (Y118) could modulate this process indirectly by interaction with CrkII or p120RasGAP, which promote the activating of GTPases and finally efficiently form leading-edge protrusion during cell migration [13]. Herein, GAEE decreased the expression of p-paxillin (Y118), we concluded that GAEE might control the molecular switches between GTP and GDP through p-paxillin-induced inhibitory influence on Rac1- and Cdc42-GEF activities, and finally affect actin assembly.

N-WASP is involved in many actin-dependent processes, such as filopodium formation and actin polymerization [53]. N-WASP transduces signals from Cdc42 to actin assembly by interacting with Arp2/3 complex and actin [54]. We found that GAEE down-regulated the expression of Cdc42, which activates N-WASP upstream, and disrupted the association between N-WASP and actin. Thus, the anti-migration effect of GAEE might be mediated via dual inhibition of the expression of upstream modulators and formation of actin assembly.

In summary, GAEE exposure suppresses cell migration and cell adhesion by inactivation of FAK and disrupting of FAK/SRC complex formation, which subsequently inhibit paxillin activation. Besides, GAEE downregulates the expression of Rho GTPases, and affects actin polymerization by disruption the association between N-WASP and Cdc42. Our results firmly suggest that GAEE presents a potential anti-metastatic property in vitro and is worthy for further testing as a potential compound for the treatment of breast cancer metastasis.

## Supporting Information

Figure S1 **Effects of GAEE on NF-κB/MMPs/uPA signaling in MDA-MB-231 cancer cells.** (A) Cells were treated with indicated concentrations of GAEE for 24 h, and the expression of MMP-2, MMP-9, uPA, and uPAR were detected. GAPDH was used as an internal control. (B) The relative densities of MMP-2, MMP-9 and uPA were determined by linear densitometric scanning to the GAPDH. Experiments were performed in triplicate and the values represented as the mean ± SEM (C) The media supernatants were collected and MMP-2, MMP-9 and uPA activities were determined by gelatin zymography and casein-plasminogen zymography, respectively. (D) MMP-2, MMP-9 and uPA activities were quantified by densitometric analysis. (E) Cells in 24-well plates were treated with GAEE (20 µg/ml) for 24 h, and immunofluorescence of NF-κB distribution (green) was detected. (F) Cells were treated with GAEE at various concentrations for 24 h, nuclear and cytosolic extracts were subjected to western blot analysis to detect the expression of NF-κB. Histone H1 and β-Actin were used as internal controls.
(TIF)
