# Supplementary figures and images for: Ganoderiol A-Enriched Extract Suppresses Migration and Adhesion of MDA-MB-231 Cells by Inhibiting FAK-SRC-Paxillin Cascade Pathway
Source: PLoS One. 2013 Oct 29;8(10):e76620. doi: 10.1371/journal.pone.0076620 (PMC3812178; doi:10.1371/journal.pone.0076620)

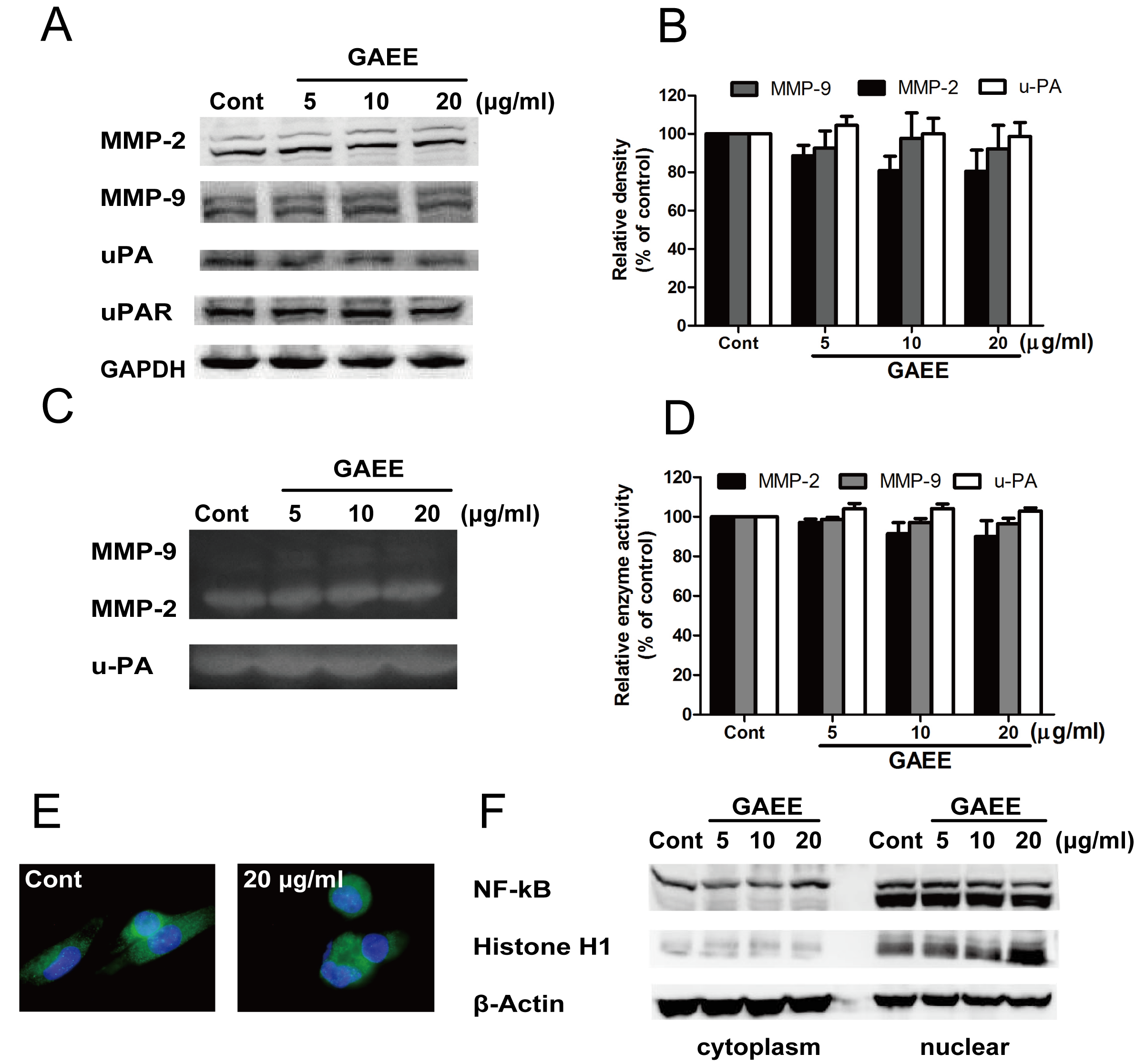

Supplement: Figure S1 — Effects of GAEE on NF-κB/MMPs/uPA signaling in MDA-MB-231 cancer cells. (A) Cells were treated with indicated concentrations of GAEE for 24 h, and the expression of MMP-2, MMP-9, uPA, and uPAR were detected. GAPDH was used as an internal control. (B) The relative densities of MMP-2, MMP-9 and uPA were determined by linear densitometric scanning to the GAPDH. Experiments were performed in triplicate and the values represented as the mean ± SEM (C) The media supernatants were collected and MMP-2, MMP-9 and uPA activities were determined by gelatin zymography and casein-plasminogen zymography, respectively. (D) MMP-2, MMP-9 and uPA activities were quantified by densitometric analysis. (E) Cells in 24-well plates were treated with GAEE (20 µg/ml) for 24 h, and immunofluorescence of NF-κB distribution (green) was detected. (F) Cells were treated with GAEE at various concentrations for 24 h, nuclear and cytosolic extracts were subjected to western blot analysis to detect the expression of NF-κB. Histone H1 and β-Actin were used as internal controls. (TIF) [file pone.0076620.s001.tif]
